# Stigmasterol Modulates Allergic Airway Inflammation in Guinea Pig Model of Ovalbumin-Induced Asthma

**DOI:** 10.1155/2017/2953930

**Published:** 2017-05-05

**Authors:** Aaron Opoku Antwi, David Darko Obiri, Newman Osafo

**Affiliations:** Department of Pharmacology, Faculty of Pharmacy and Pharmaceutical Sciences, College of Health Sciences, Kwame Nkrumah University of Science & Technology (KNUST), Kumasi, Ghana

## Abstract

We explored the potential benefits of stigmasterol in the treatment of asthma, an airway disorder characterized by immune pathophysiology and with an ever-increasing worldwide prevalence. We assessed the modulatory effect of the intraperitoneal administration of stigmasterol on experimentally induced airway inflammation in guinea pigs. The effect of stigmasterol on inflammatory cell proliferation, oxidative stress, lung histopathology, and remodeling was investigated. The results showed significant suppressive effects on ovalbumin-induced airway inflammatory damage. Stigmasterol at 10–100 mg/kg reduced proliferation of eosinophils, lymphocytes, and monocytes while reducing peribronchiolar, perivascular, and alveolar infiltration of inflammatory cells. Histopathology revealed stigmasterol maintained lung architecture and reversed collagen deposition, an index of lung remodeling. Overexpression of serum vascular cell adhesion molecule-1 (VCAM-1) and ovalbumin-specific immunoglobulin E (OVA sIgE) elicited by ovalbumin sensitization and challenge was significantly controlled with stigmasterol. Taken together, stigmasterol possessed significant antiasthmatic properties and had suppressive effects on key features of allergen-induced asthma.

## 1. Introduction

Stigmasterol, a naturally occurring steroid alcohol, belongs to a larger class of plant compounds called phytosterols [1] which are widely distributed in foods of plant origin [2] and popular medicinal plants throughout the world [3–5]. Phytosterols have established and emerging health benefits including lipid lowering, anticancer, anti-inflammatory, and antiallergic effects [6]. Several in vivo and in vitro studies have shown wide reaching anti-inflammatory actions as possible explanations for the apparent lipid lowering and antiatherogenic effects [7]. Demonstration of antiarthritic and anti-inflammatory actions [8, 9] for stigmasterol suggests some immunomodulatory effects, but there still exists a significant knowledge gap with regard to the extent and specifics of its role in immune and inflammatory disorders such as asthma.

Asthma is a chronic pulmonary disorder associated with airway hyperresponsiveness (AHR), inflammation, and airway obstruction. The pathophysiology of asthma is characterized by severe inflammatory cell activation and accumulation, airway muscle hypertrophy, submucosal fibrosis, and excessive mucus production resulting in permanent airway remodeling [10]. In allergic asthma, immunoglobulin E (IgE) type of antibodies is produced when cognate antigens also called allergens sensitize patients on the first exposure. These antibodies remain in circulation in the blood or become attached to mast cells of the nasal or bronchial tissues and basophils. When such subjects are reexposed to the same antigen, cross-linking of bound immunoglobulin E (IgE) to surface receptors occurs [11]. At the molecular level, this antigen–antibody reaction in the early phase causes degranulation of the lung mast cells with the release of mediators such as histamine, 5-hydroxytryptamine, prostaglandins, the cysteinyl leukotrienes (LTB_4_, LTC_4_ and LTD_4_), and cytokines such as the interleukins IL-4, IL-5, and IL-13 [12]. Elias et al. [13] report that these mediators of allergy sustain the late or delayed phase of asthma and they activate additional inflammatory cells such as eosinophils, basophils, leucocytes, and alveolar macrophages to release more of the LTs and ILs. Recent advances in medicine notwithstanding, asthma is responsible for a yearly death toll of about 250,000. This has ultimately imposed a global financial burden of about $300–1300 per patient, annually in developed countries, and with increasing trends observed in low- to middle-income countries, these values are expected to increase worldwide [14]. New research and medications designed to tackle specific arms of the underlying pathophysiology have emerged [15, 16] in an attempt to address the current gap in knowledge and limitations in therapy, respectively.

The search for novel medications for asthma spans across synthetic molecules, molecular interventions, and alternatives from natural sources. Particular interest in the latter has taken center stage, with some interesting findings already reported from both experimental and clinical investigations [17–19].

In this study, we investigate the potential benefits of stigmasterol in the treatment of asthma. We assess its possible anti-inflammatory or immunomodulatory effects in ovalbumin-induced asthma in guinea pig model of inflammation.

## 2. Material and Methods

### 2.1. Materials

#### 2.1.1. Chemicals and Reagents

Stigmasterol (98%), ovalbumin (OVA), and dexamethasone were obtained from Sigma Aldrich (St. Louis, USA). Guinea pig VCAM-1 and OVA sIgE ELISA quantification kits were purchased from MLBio Biotechnology Company Limited (Shanghai, China).

#### 2.1.2. Animals

Guinea pigs (300–350 g) of either sex were obtained from Noguchi Memorial Institute for Medical Research, Legon, Ghana. Animals were kept under standard temperature and humidity conditions (temperature 23 ± 2°C with a 12 h light-dark cycle) at the Animal House facility of the Department of Pharmacology, Faculty of Pharmacy and Pharmaceutical Sciences, KNUST, and allowed access to commercial chow and distilled water ad libitum. All protocols used in this study were approved by the Faculty of Pharmacy Ethics Committee, and animal handling was done in compliance with the National Institute of Health Guidelines for Care and Use of Animals.

### 2.2. Methods

#### 2.2.1. Ovalbumin-Induced Asthma: Sensitization and Challenge

Five groups (*n* = 5) of guinea pigs (300–350 g) were sensitized by intraperitoneal injection of 100 *μ*l OVA solution (2 mg ovalbumin emulsified in 10 mg aluminium hydroxide (AlOH_3_) dissolved in 10 ml normal saline (0.9% *w*/*v* NaCl) at the start of the experiment. A booster dose of 100 *μ*l solution (1 mg ovalbumin dissolved in saline) was administered intraperitoneally on day 14. From day 21 to day 30, sensitized guinea pigs were challenged with aerosolized ovalbumin (1% *w*/*v* dissolved in phosphate-buffered saline, PBS) daily for 10 min. Naïve animals (*n* = 5) were sham-sensitized with 100 *μ*l normal saline and challenged with PBS only. Before each challenge, normal saline (10 ml/kg, p.o.), stigmasterol (10, 50, 100 mg/kg, i.p.) or dexamethasone (3 mg/kg, p.o.) respectively, was administered to the naïve and test groups 30 min after i.p. or 1 h after oral administration. Guinea pigs were subjected to the following tests.

*(1) Haematology and Serum Analysis*. 24 h after the last OVA exposure, all animals were sacrificed with an overdose of ether and immediately bled by dissection of the jugular vein. Blood was collected into EDTA tubes for differential blood cell count using a haemocytometer and into sterile capillary tubes for serum analysis, respectively. Blood collected into sterile capillary tubes was allowed to clot and centrifuged for 15 min at 1000 rpm. Aliquots of serum were collected into Eppendorf tubes and stored at −70°C. Serum levels of vascular cell adhesion molecule-1 (VCAM-1) and ovalbumin-specific immunoglobulin E (OVA sIgE) were quantified using enzyme-linked immunosorbent assay (ELISA) kits according to instructions of the manufacturers.

*(2) Bronchoalveolar Lavage Fluid (BALF) Collection and Analysis*. Immediately after blood collection, the trachea was carefully exposed avoiding contamination of luminal contents with blood and damage to the lung tissues. The trachea was cannulated and bronchoalveolar fluid was collected by aspiration. The lung tissues were flushed with 3 × 5 ml portions of PBS and aspirated after gentle massage. Recovered fluid was centrifuged at 1000 rpm for 10 min at 4°C. The supernatant was collected, protein concentration determined with the Bradford method, stored at −70°C, and when needed subjected to analysis for the following oxidative stress markers.

*(3) Malondialdehyde (MDA)*. MDA was measured as a product of lipid peroxidation by the method of Heath and Packer [20]. Briefly, 1 ml BALF was added to a 3 ml mixture of trichloroacetic acid, TCA (20%), and thiobarbituric acid, TBA (0.5%), heated at 95°C for 30 min and immediately cooled and centrifuged at 5000 rpm for 10 min. 200 *μ*l aliquots of supernatant were pipetted into 96-well plates in triplicate, and absorbance was read at 532 nm and 600 nm, respectively, with a Synergy H1 Hybrid Reader spectrophotometer (BioTek Technologies, Winooski, VT, USA) to correct for nonspecific absorbance. MDA concentration (nmol/mg protein) was calculated with its molar extinction coefficient of 1.56 × 10^−5^ M^−1^ cm^−1^ with the equation

(1) $$\begin{array}{c} nmol\;MDA\;per\;mg\;protein=\left[\frac{{Absorbance}_{532\;nm}-{Absorbance}_{600\;nm}}{1.56\times 10^{5}\times total\;protein}\;\right]\times 10^{6}. \end{array}$$

*(4) Reduced Glutathione (GSH)*. GSH levels were determined by a method earlier described by Ellman [21]. Briefly, 100 *μ*l BALF aliquot was mixed with 2.4 ml EDTA (0.02 M) at 4°C for 10 min. 2 ml distilled water and 500 *μ*l TCA (50%) were added and centrifuged at 3000 rpm for 5 min. 1 ml of the supernatant, 50 *μ*l 5,5′-dithio-*bis-2-*nitro benzoic acid, DTNB (10 mM), and 2 ml Tris buffer (0.4 M, pH 8.9) were added. The absorbance was read within 5 min of DTNB addition at 412 nm against a blank (reagents only) with a Synergy H1 Hybrid Reader spectrophotometer (BioTek Technologies, Winooski, VT, USA). The final sulfhydryl concentration was interpolated from a standard curve with the equation  *y* = 0.0004 *x* + 0.0026, where *x* is the absorbance at 412 nm.

*(5) Superoxide Dismutase (SOD)*. SOD activity was estimated with the modified method of Misra and Fridovich [22]. Briefly, 500 *μ*l tissue supernatant was treated with 150 *μ*l ice-cold chloroform and 750 *μ*l ethanol (96% *v*/*v*), vortexed for 1 min and then centrifuged at 2000 rpm for 20 min. 500 *μ*l portion of the supernatant, 500 *μ*l EDTA (0.6 mM), and 1 ml carbonate bicarbonate buffer (0.1 M, pH 10.2) were added. The reaction was initiated by the addition of 50 *μ*l adrenaline (1.3 mM). Absorbance was measured with a Synergy H1 Hybrid Reader spectrophotometer (BioTek Technologies, Winooski, VT, USA) at 480 nm against a blank. Activity of SOD, measured as the quantity of the enzyme required to inhibit the auto-oxidation of adrenaline, was calculated using the equation

(2) $$\begin{array}{c} \%\;inhibition=\left[\frac{{Absorbance}_{\left(test\right)}-{Absorbance}_{\left(blank\right)}}{{Absorbance}_{test}}\right]\times 100. \end{array}$$

SOD level was expressed in units per mg protein, where 1 unit of enzyme activity is the quantity of enzyme required to prevent the auto-oxidation of adrenaline at 25°C, and calculated with the equation

(3) $$\begin{array}{c} units\;of\;SOD\;activity/mg\;protein=\left[\frac{\%\;inhibition\;}{\;50\times weight\;of\;protein}\right]\times 100. \end{array}$$

*(6) Catalase (CAT)*. The method described by Sinha [23] with slight modifications was used. Briefly, 100 *μ*l aliquot of tissue supernatant, 1 ml phosphate buffer (0.01 M, pH 7.0), and 400 *μ*l H_2_O_2_ (1.18 M) were added, and the mixture was incubated at room temperature for 5 min. The reaction was halted by adding 2 ml of a 3 : 1 mixture of glacial acetic acid and dichromate (5%). Absorbance was measured at 620 nm with a Synergy H1 Hybrid Reader spectrophotometer (BioTek Technologies, Winooski, VT, USA). One unit of catalase activity, defined as the amount of enzymes that degrades 1 mmol H_2_O_2_ per min at 25°C and pH 7.0, was expressed in terms of its molar extinction coefficient, 39.4 M^−1^ cm^−1^.

(4) $$\begin{array}{c} mUnit\;CAT/mg\;protein=\left[\frac{{Absorbance}_{620\;nm}}{\;3.94\times weight\;of\;protein}\right]\times 1000. \end{array}$$

#### 2.2.2. Histology

24 h after the last OVA exposure, the lung tissues were carefully removed and fixed in 10% formalin. Tissues were serially dehydrated in increasing concentrations of ethanol, cleared in xylene in a TP 1020 Tissue processor (Leica Biosystems, Wetzlar, Germany), and embedded in paraffin using a Leica EG 1160 Embedding machine (Leica Biosystems, Wetzlar, Germany). Transverse sections of 3 *μ*m were cut with a Leica RM 2125 Microtome (Leica Biosystems, Wetzlar, Germany), deparaffinized, and hydrated to distilled water. Tissue sections were stained appropriately for either airway inflammatory cell infiltration or collagen deposition and observed under light microscope (Leica DM2500 M). Quantitative analyses were performed with ImageJ analysis tool (version 1.50i).

*(1) Airway Inflammatory Cell Infiltration*. Sections were stained with hematoxylin and eosin (H & E) stain. A method previously described by Zare et al. [24] with modifications was used to assess the degree of airway inflammatory cell infiltration with a brief scoring system as follows: 0, no cell; 1, a few cells; 2, a ring of cells 1 cell layer deep; 3, a ring of cells 2–4 cell layers deep; and 4, a ring of cells > 4 cell layers deep in the peribronchiolar and perivascular regions. Alveolar cell infiltration was assessed as follows: 0, no infiltrate or widening septa; 1, few infiltrates with widening septa; 2; obvious infiltrates with widening septa; and 3, filled alveolar air spaces with thickened septa. Scores for peribronchiolar, perivascular, and alveolar cell infiltration were summed into an 11-point composite score.

*(2) Assessment of Collagen Deposition*. Sections were stained with Masson's trichrome stain. Remodeling was assessed by measuring the total length of the basement membrane of selected bronchioles from each treatment and the respective peribronchiolar fibrotic region (stained blue). The degree of fibrosis was quantified as the mean area of collagen deposition per unit length of the basement membrane. For each guinea pig, 5 random sections from the left lower lung were selected. Five to seven average-sized bronchioles from each section were analyzed, and the average scores for each group were calculated.

### 2.3. Statistical Analysis

All results are presented as mean ± SEM. Data analysis was done using one-way analysis of variance (ANOVA). Multiple comparisons between the treatment groups were performed using Dunnet's post hoc test. All statistical analyses were done using GraphPad for Windows version 6 (GraphPad Prism Software, San Diego, USA).

## 3. Results

### 3.1. Haematology and Serum Analysis

#### 3.1.1. Effect of Stigmasterol on Inflammatory Cell Count in Blood

**Figure 1:**
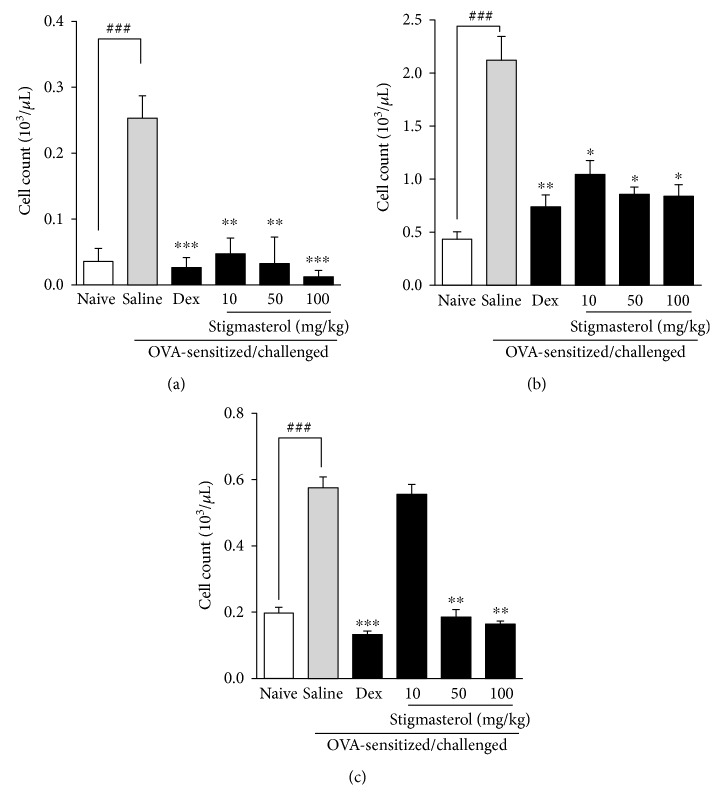
Effect of stigmasterol on inflammatory cell count in blood. Guinea pigs were sensitized and challenged with ovalbumin as described in the methods. Animals received either saline, dexamethasone, or stigmasterol 1 h prior to each challenge. Naïve controls received normal saline only. Animals were sacrificed 24 h after the last challenge, and blood was collected for counts of eosinophil (a), lymphocyte (b), and monocyte (c). Data is expressed as cell count (10^3^/*μ*L) ± SEM (*n* = 5). ^∗∗∗^*P* < 0.001, ^∗∗^*P* < 0.01, and ^∗^*P* < 0.5 as compared to saline-treated control; ^###^*P* < 0.001 and ^##^*P* < 0.01 as compared to naïve control using one-way ANOVA followed by Dunnet's post hoc test.

Ovalbumin challenge of previously OVA-sensitized guinea pigs was characterized by significant increase of blood eosinophils, lymphocytes, and monocytes relative to the naïve control animals (Figures 1(a), 1(b), and 1(c)). The saline-treated OVA-sensitized and challenged groups recorded, respectively, 7-, 5-, and 3-fold increases in mean number of eosinophils, lymphocytes, and monocytes compared to their respective naïve control groups, and these were significantly reduced by dexamethasone (Figures 1(a), 1(b), and 1(c)). Stigmasterol administered at 10, 50, and 100 mg/kg significantly reduced the cell proliferative effect induced by ovalbumin challenge from 0.25 ± 0.01 to 0.05 ± 0.01, 0.03 ± 0.02, and 0.02 ± 0.01, respectively, for eosinophils (Figure 1(a)). Similarly elevated blood lymphocyte count of 2.12 ± 0.09 were significantly reduced by stigmasterol treatment to 1.05 ± 0.05, 0.86 ± 0.03 and 0.84 ± 0.04, respectively (Figure 1(b)). For the monocytes, while significantly reduced numbers were counted from a control of 0.58 ± 0.03 to 0.19 ± 0.02 and 0.16 ± 0.01, respectively, for 50 and 100 mg/kg stigmasterol treatment, the value of 0.56 ± 0.03 obtained for the 10 mg/kg treatment was not significant (Figure 1(c)).

#### 3.1.2. Effect of Stigmasterol on Serum Vascular Cell Adhesion Molecule-1 (VCAM-1) Levels

**Figure 2:**
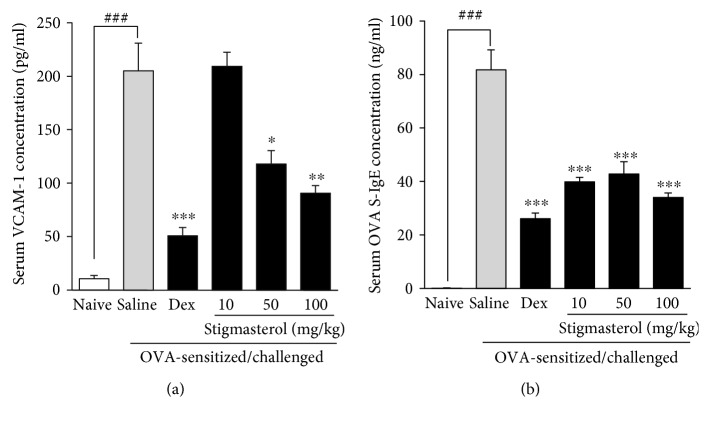
Effect of stigmasterol on serum vascular cell adhesion molecule-1 (VCAM-1) and serum OVA-specific immunoglobulin E (OVA sIgE). Guinea pigs were sensitized and challenged with ovalbumin as described in the methods. Animals received either saline, dexamethasone, or stigmasterol 1 h prior to each challenge. Naïve controls received normal saline only. 24 h after the last challenge, animals were bled by dissection of the jugular vein. Blood collected was allowed to clot and centrifuged at 1000 rpm for 15 min. Serum vascular cell adhesion molecule-1, VCAM-1 (a), and serum OVA-specific immunoglobulin E, OVA sIgE (b), levels were quantified with sandwich ELISA. Data is expressed as VCAM-1 or OVA sIgE concentration (pg/ml) ± SEM (*n* = 5). ^∗∗∗^*P* < 0.001, ^∗∗^*P* < 0.01, and ^∗^*P* < 0.5 as compared to saline-treated control; ^###^*P* < 0.001 as compared to naïve control using one-way ANOVA followed by Dunnet's post hoc test.

Serum analysis showed a significantly increased mean expression of soluble vascular cell adhesion molecule-1 (VCAM-1) in the saline-treated OVA-sensitized and challenged group to 205.20 ± 25.82 × 10^−12^ g/ml from a mean value of 10.58 ± 3.18 × 10^−12^ g/ml for the naïve control group (Figure 2(a)). As expected, dexamethasone (3 mg/kg) significantly reduced the increased mean expression of VCAM-1 to 50.97 ± 7.52 × 10^−12^ g/ml. Stigmasterol 50 and 100 mg/kg demonstrated inhibitory effects by significantly reducing the mean expression of VCAM-1 to 118 ± 12.54 × 10^−12^ g/ml and 90.75 ± 7.12 × 10^−12^ g/ml, respectively, when compared to the saline-treated OVA-sensitized and challenged control group. No significant inhibition was however observed with stigmasterol administered at 10 mg/kg with an expression level of 209.40 ± 13.11 × 10^−12^ g/ml (Figure 2(a)).

#### 3.1.3. Effect of Stigmasterol on Serum OVA-Specific Immunoglobulin E (OVA sIgE) Levels

The mean OVA sIgE was significantly increased to 81.75 ± 7.5 × 10^−9^ g/ml in the saline-treated OVA-sensitized and challenged group compared to the naïve control animals with values below detectable levels (Figure 2(b)). OVA sIgE levels in dexamethasone-treated animals were significantly reduced to 26.10 ± 2.08 × 10^−9^ g/ml as compared to the saline-treated OVA-sensitized and challenged group (Figure 2(b)). Stigmasterol significantly reduced ovalbumin-elicited serum levels of OVA-specific immunoglobulin E to 39.83 ± 1.71 × 10^−9^ g/ml, 42.79 ± 4.59 × 10^−9^ g/ml, and 34.02 ± 1.65 × 10^−9^ g/ml, respectively, at 10, 50, and 100 mg/kg relative to the saline-treated OVA-sensitized and challenged control group (Figure 2(b)).

### 3.2. Bronchoalveolar Lavage Fluid (BALF) Analysis

#### 3.2.1. Effect of Stigmasterol on BALF Oxidative Stress Markers

**Figure 3:**
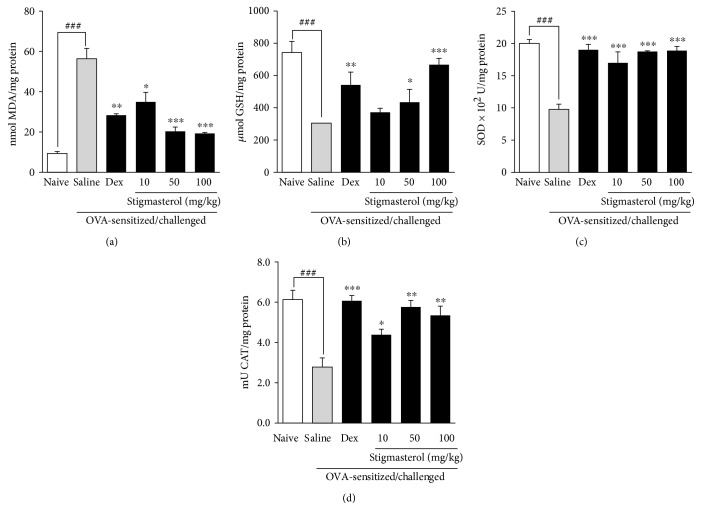
Effect of stigmasterol on BALF oxidative stress markers. Guinea pigs were sensitized and challenged with ovalbumin as described in the methods. Animals received either saline, dexamethasone, or stigmasterol 1 h prior to each challenge. Naïve controls received normal saline only. Bronchoalveolar fluid was collected by aspiration 24 h after the last ovalbumin challenge and centrifuged for 1000 rpm for 10 min. The supernatant was analyzed quantitatively for level of malondialdehyde (a), reduced glutathione (b), superoxide dismutase (c), and catalase (d). Data is expressed as mean concentration/mg protein ± SEM (*n* = 5). ^∗∗∗^*P* < 0.001, ^∗∗^*P* < 0.01, and ^∗^*P* < 0.05 as compared to the saline-treated group, and ^###^*P* < 0.001 as compared to the naïve group using one-way ANOVA followed by Dunnet's post hoc test.

Analysis of supernatant showed an antioxidant profile consistent with severe inflammation. Levels of malondialdehyde (MDA), a direct product of lipid peroxidation, were significantly elevated to 56.39 ± 5.15 nmol/mg protein in the saline-treated OVA-sensitized and challenged animals relative to 9.30 ± 1.01 nmol/mg protein in the naïve animals (Figure 3(a)). This evident oxidative stress induced by ovalbumin challenge was significantly mitigated by both dexamethasone and stigmasterol. Administered at 3 mg/kg, dexamethasone suppressed the elevated MDA level to 28.36 ± 0.65 nmol/mg protein while stigmasterol at 10, 50, and 100 mg/kg significantly suppressed the same to 34.98 ± 4.72, 20.32 ± 2.08, and 19.28 ± 0.52 nmol/mg protein, respectively (Figure 3(a)). Analysis of BALF from the saline-treated OVA-sensitized and challenged group showed significant depletion of the markers reduced glutathione (GSH), superoxide dismutase (SOD), and catalase (CAT) with dexamethasone significantly increasing their levels (Figures 3(b), 3(c), and 3(d)). The mean GSH levels of 742.40 ± 26.23 *μ*mol/mg protein in naïve guinea pigs was a significantly reduced to 304.60 ± 4.04 *μ*mol/mg protein in the saline-treated OVA-sensitized and challenged animals. Dexamethasone as expected elevated it to 541.70 ± 29.87 *μ*mol/mg protein (Figure 3(b)). Stigmasterol 50 and 100 mg/kg significantly maintained GSH levels at 434.20 ± 30.19 and 667.40 ± 15.14 *μ*mol/mg protein, respectively, while the mean value of 372.10 ± 9.65 *μ*mol/mg protein recorded in the 10 mg/kg stigmasterol-treated animals was however not statistically different from saline-treated ovalbumin-sensitized and challenged control (Figure 3(b)). Superoxide dismutase level was significantly reduced to 9.78 ± 0.28 × 10^2^ U/mg protein in the saline-treated OVA-sensitized and challenged animals relative to 20.02 ± 0.21 × 10^2^ U/mg protein in the naïve animals (Figure 3(c)). When treated with dexamethasone, a significantly elevated SOD level of 19.02 ± 0.34 × 10^2^ U/mg protein was attained relative to the saline-treated OVA-sensitized and challenged animals while stigmasterol at 10, 50, and 100 mg/kg significantly elevated mean SOD to 16.99 ± 0.70, 18.74 ± 0.05, and 18.89 ± 0.27 × 10^2^ U/mg protein, respectively (Figure 3(c)). A similar observation was made for CAT levels (Figure 3(d)). On OVA sensitization and challenge, saline-treated animals presented with 2.79 ± 0.45 mU/mg protein which was significantly different from 6.13 ± 0.46 mU/mg protein in the naïve animals. Dexamethasone significantly elevated CAT level to 6.08 ± 0.26 mU/mg protein, while the test drug stigmasterol at 10, 50, and 100 mg/kg significantly elevated CAT levels to 4.38 ± 0.28, 5.77 ± 0.32, and 5.35 ± 0.46 mU/mg protein, respectively (Figure 3(d)).

### 3.3. Histology

#### 3.3.1. Effect of Stigmasterol on Inflammatory Cell Infiltration

**Figure 4:**
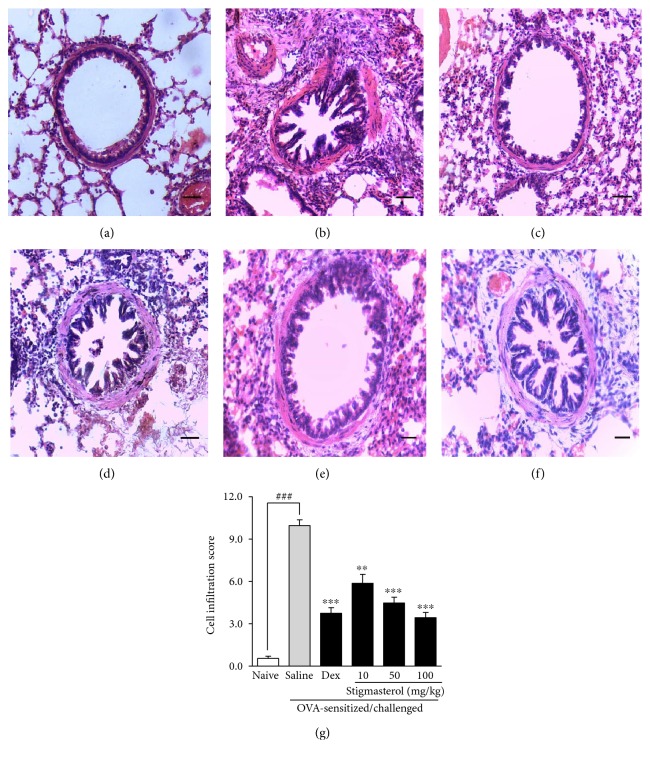
Effect of stigmasterol on inflammatory cell infiltration. Guinea pigs were sensitized and challenged with ovalbumin as described in the methods. Animals received either saline, dexamethasone, or stigmasterol 1 h prior to each challenge. Naïve controls received normal saline only. Animals were sacrificed 24 h after the last ovalbumin challenge. The lungs were excised, fixed, and embedded in paraffin. 3 *μ*m sections were stained with H&E to assess cell infiltration in naïve (a), saline (b), and dexamethasone (c) and 10, 50, and 100 mg/kg stigmasterol-treated animals (d–f). Degree of infiltration was quantified using an infiltration score described by Zare et al. [24] with slight modifications (g). Data is expressed as mean cell infiltration score ± SEM (*n* = 5). ^∗∗∗^*P* < 0.001 and ^∗∗^*P* < 0.01 as compared to the saline-treated group and ^###^*P* < 0.001 as compared to the naïve group using one-way ANOVA followed by Dunnet's post hoc test.

Lung architecture in naïve animals was consistent with normal guinea pig lung structure. Alveolar spaces were clear with little or no accumulation of cells around the bronchioles (Figure 4(a)). Ovalbumin challenge in the previously sensitized animals induced severe and extensive infiltration of lymphocytes, eosinophils, and monocytes, forming thick cuffs of cells around the bronchioles, blood vessels, and alveolar septa in the saline-treated OVA-sensitized and challenged group (Figure 4(b)). Treatment with dexamethasone 3 mg/kg (Figure 4(c)) as well as stigmasterol 10–100 mg/kg (Figures 4(d), 4(e), and 4(f)) reversed these features and presented less cellular congestion and alveolar septa thickening. These effects were quantified into a composite cell infiltration score by a method earlier described. A cell infiltration score of 9.95 ± 0.40 was recorded for the saline-treated ovalbumin-sensitized and challenged group representing a significant increase when compared to 0.55 ± 0.16 for the naïve group (Figure 4(g)). Compared to the untreated asthma group, dexamethasone exhibited a significantly reduced cell infiltration score of 3.75 ± 0.39. Stigmasterol presented a significantly different dose-dependent reduction of ovalbumin-induced cell infiltration when compared to the normal saline-treated ovalbumin-sensitized and challenged group, with scores of 5.86 ± 0.64, 4.47 ± 0.40, and 3.43 ± 0.37, respectively, at the doses of 10, 50, and 100 mg/kg (Figure 4(g)).

#### 3.3.2. Effect of Stigmasterol on Collagen Deposition

**Figure 5:**
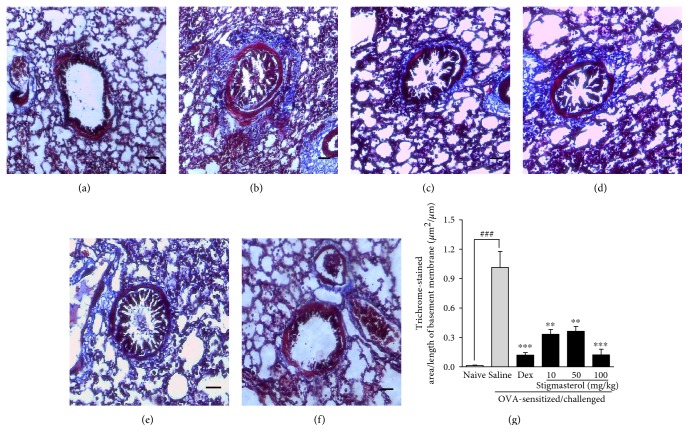
Effect of stigmasterol on inflammatory collagen deposition. Guinea pigs were sensitized and challenged with ovalbumin as described in the methods. Animals received either saline, dexamethasone, or stigmasterol 1 h prior to each challenge. Naïve controls received normal saline only. Animals were sacrificed 24 h after the last ovalbumin challenge. The lungs were excised, fixed, and embedded in paraffin. 3 *μ*m sections were stained with Masson's trichrome solution to assess collagen deposition in naïve (a), saline (b), and dexamethasone (c) and 10, 50, and 100 mg/kg stigmasterol-treated animals (d–f). Morphometric analysis was performed to quantify the extent of collagen deposition (g). Data is expressed as mean collagen deposition index ± SEM (*n* = 5). ^∗∗∗^*P* < 0.001 and ^∗∗^*P* < 0.01 as compared to the saline-treated group and ^###^*P* < 0.001 as compared to the naïve group using one-way ANOVA followed by Dunnet's post hoc test.

Subepithelial collagen deposition (blue stain) was significantly pronounced in the saline-treated OVA-sensitized and challenged group mostly in the perivascular and peribronchiolar regions (Figure 5(b)), a feature absent in the lung tissues of the naïve animals (Figure 5(a)). Subepithelial collagen deposition was significantly reduced in dexamethasone-treated guinea pigs (Figure 5(c)). Similar reductions were noted with the stigmasterol-treated groups when compared to the saline-treated OVA-sensitized and challenged group (Figures 5(d), 5(e), and 5(f)). Morphometric analyses confirmed this observation. Quantitative measurements showed a collagen deposition index (stained area/per unit basement membrane length) of 1.02 ± 0.16 *μ*m^2^/*μ*m in the saline-treated OVA-sensitized and challenged group relative to 0.01 ± 0.01 *μ*m^2^/*μ*m in the naïve control group (Figure 5(g)). Dexamethasone was significantly effective in reducing the fibrotic region to 0.12 ± 0.03 *μ*m^2^/*μ*m. Stigmasterol at the doses of 10, 50, and 100 mg/kg recorded indices of 0.33 ± 0.05 *μ*m^2^/*μ*m, 0.36 ± 0.05 *μ*m^2^/*μ*m, and 0.12 ± 0.06 *μ*m^2^/*μ*m, respectively, that were significant relative to the saline-treated OVA-sensitized and challenged group (Figure 5(g)).

## 4. Discussion

We explored the effects of stigmasterol on chronic airway inflammation induced by aerosolized ovalbumin and investigated its potential inhibitory effect on inflammatory features triggered by repeated challenge with ovalbumin in previously sensitized guinea pigs noting the possible mechanisms involved in the inhibition.

Cell infiltration into the lung tissues and alveolar fluids, elevation of inflammatory cells in blood, and changes to lung histology are features largely consistent with asthma [25]. The ovalbumin-induced asthma model has been employed extensively and established to have characteristics that represent human asthma [26]. In this model, ovalbumin serves as the source of allergen. After sensitization and aerosol challenge, a Type 1 immune response is triggered. A Th2-skewed response which is characterized by Th2 cytokines such as IL-4, IL-5, and IL-13 tends to mediate production of ovalbumin-specific IgE by B cells and recruitment of eosinophils, mast cells, and other inflammatory cells. Proinflammatory cytokine participation mainly by IL-1, IL-6, and tumor necrosis factor alpha (TNF*α*) further intensifies the inflammatory response [27]. Consequently, there is a direct tissue injury and high cellular activity which invariably initiates production of reactive oxygen species (ROS) resulting in damage to macromolecular structures [28]. The extent of oxidative damage can be measured by levels of MDA, a product of lipid peroxidation, and respective levels of the body's enzyme (SOD, catalase) and nonenzyme (GSH) antioxidant factors [29, 30]. This model is thus appreciably predictive in the investigation of potential antiasthmatic agents.

In this study, we could show that stigmasterol inhibited early phase immune responses to allergen exposure. Analysis of serum collected from guinea pigs 24 h after the last ovalbumin exposure showed elevated levels of ovalbumin-specific immunoglobulin E, OVA sIgE in saline-treated OVA-sensitized and challenged controls and significantly reduced in stigmasterol-treated animals. Elevated blood inflammatory cell count induced by OVA sensitization and challenge in the saline-treated animals was also suppressed by stigmasterol. Similar to dexamethasone, stigmasterol could significantly control eosinophil, lymphocyte, and monocyte proliferation. In asthma, the proliferation of blood borne inflammatory cells and their subsequent migration into airway tissue drives epithelial tissue damage caused by chemical and inflammatory mediator release, leading to severe inflammation. Indeed previous studies have established a direct link between Th2 cell control and a good asthma prognosis [31].

Prior to tissue invasion, inflammatory cell movement and subsequent adherence to endothelial cells are mediated by several adhesion molecules. VCAM-1, identified as a major adhesion molecule in this process, is shed from cytokine-activated endothelial cells to promote subsequent leucocyte attachment [32]. Soluble or circulating forms of VCAM-1 were upregulated in the saline-treated asthmatic control animals. We could demonstrate significantly reduced VCAM-1 levels in the stigmasterol-treated animals at 50 and 100 mg/kg suggesting that stigmasterol may have an overall suppressive role on cell-mediated lung tissue damage and eventual remodeling.

Significant correlation between asthma severity and either systemic or airway oxidative stress has been established [30]. Cellular and tissue damage associated with asthma leads to massive production of reactive oxygen species [33]. Reduction in *β*-adrenergic function in the lungs, increased sensitivity of airway smooth muscle, tracheal smooth muscle contraction, and mucus production have been attributed to the role of ROS [34]. It is therefore not surprising that several studies have established a positive relationship between lung function and antioxidant intake. Levels of oxidative stress markers in breath condensates [35] and bronchoalveolar fluids [36] have been used to predict asthma severity in both animal and clinical studies. From analysis of bronchoalveolar lavage fluid, BALF, it was observed that treatment with stigmasterol inhibited oxidative stress evidenced in the preservation of the lung tissue antioxidant capacity. Markers such as reduced glutathione (GSH), superoxide dismutase (SOD), and catalase (CAT) in BALF were significantly elevated in the stigmasterol-treated animals compared to asthmatic control animals. Saline-treated asthmatic control animals relative to the stigmasterol-treated animals presented with significantly higher levels of MDA, a product of lipid peroxidation and a positive indicator of oxidative stress. Results from antioxidant studies showed that potentially damaging processes such as lipid peroxidation and superoxide anion-mediated free radical generation were abated. This apparent antioxidant effect of stigmasterol is consistent with some previous studies reporting antioxidant activity of stigmasterol in both in vitro and in vivo assays [37, 38].

Hematoxylin and eosin (H&E) staining revealed excessive infiltration of inflammatory cells, mostly eosinophils and lymphocytes, in the ovalbumin challenged groups compared to that in untreated naïve animals. Lung sections of saline-treated OVA-sensitized and challenged animals showed high cellularity especially in peribronchiolar and perivascular regions. Dexamethasone and stigmasterol lowered cell accumulation in the peribronchiolar, perivascular, and alveolar regions, obtaining lower cell infiltration scores compared to the saline-treated OVA-sensitized and challenged asthmatic control guinea pigs. Stigmasterol treatment was associated with less congestion, sparsely distributed inflammatory cells in the alveolar region, and reduced thickening of alveolar septa. Persistent uncontrolled airway inflammation and cell infiltration lead to a cycle of tissue damage and repair eventually causing permanent damage to the lung tissues referred to as lung remodeling. It is associated with airway smooth muscle thickening, epithelial and goblet cell hyperplasia, basement membrane thickening, and collagen deposition [39]. In this study, we assessed collagen deposition as an index for lung remodeling and tissue fibrosis employing the Masson's trichrome stain. As expected, sections from the saline-treated OVA-sensitized and challenged asthmatic control animals showed extensive areas of collagen-positive staining especially around the bronchioles and blood vessel but significantly reduced in sections from stigmasterol-treated animals. A collagen deposition index showed all three doses of stigmasterol effective in suppressing lung remodeling.

Taken together, our data demonstrates for the first time that stigmasterol suppresses airway inflammation and remodeling by inhibiting allergen-induced immunoglobulin E-mediated responses and also abolishes VCAM-1-aided cellular migration into the lung tissues. Again, we show in part here that stigmasterol controls oxidative stress and preserves lung tissue antioxidant capacity, and this mechanism is a key factor responsible for its anti-inflammatory action.

## 5. Conclusion

Stigmasterol inhibits OVA-induced asthma in guinea pigs and has potential as a molecule of interest for the treatment of asthma.
